# Evaluation of internal reliability in the presence of inconsistent responses

**DOI:** 10.1186/1477-7525-8-27

**Published:** 2010-03-12

**Authors:** Daniel YT Fong, S Y Ho, T H Lam

**Affiliations:** 1 School of Nursing, Li Ka Shing Faculty of Medicine, The University of Hong Kong, Pokfulam Road, Hong Kong; 2 Department of Community Medicine and School of Public Health, Li Ka Shing Faculty of Medicine, The University of Hong Kong, Pokfulam Road, Hong Kong

## Abstract

**Background:**

We aimed to assess the impact of inconsistent responses on the internal reliability of a multi-item scale by developing a procedure to adjust Cronbach's alpha.

**Methods:**

A procedure for adjusting Cronbach's alpha when there are inconsistent responses was developed and used to assess the impact of inconsistent responses on internal reliability by evaluating the standard Chinese 12-item Short Form Health Survey in adolescents.

**Results:**

Contrary to common belief, random responses may inflate Cronbach's alpha when their mean differ from that of the true responses. Fixed responses inflate Cronbach's alpha except in scales with both positive and negative polarity items. In general, the bias in Cronbach's alpha due to inconsistent responses may change from negative to positive with an increasing number of items in a scale, but the effect of additional items beyond around 10 becomes small. The number of response categories does not have much influence on the impact of inconsistent responses.

**Conclusions:**

Cronbach's alpha can be biased when there are inconsistent responses, and an adjustment is recommended for better assessment of the internal reliability of a multi-item scale.

## Background

Internal reliability is an attribute of a multi-item scale that refers to the extent to which items in the scale are related; it is very often evaluated to assess the reliability of patient-reported outcomes (PROs). The most common measure of internal reliability reported in psychometric studies of PROs is Cronbach's alpha [1], but unfortunately, it can be biased by the presence of inconsistent responses.

Inconsistent responding occurs when respondents complete a questionnaire without comprehending the items, typically in self-reported questionnaires when the participants are unmotivated or the questions are sensitive [2]. Inconsistent responses are classified as random, when responses are given unsystematically, or fixed, when the same response is given to all items [3]. Although the literature has not stipulated the impact of inconsistent responses on internal reliability, fixed responses by their nature would result in high association among the responses of the associated items and thus inflate the observed reliability in scales whose items have the same polarity. They can also diminish it in scales when that is not the case as the association among the item responses would be lower. Moreover, a substantial number of random responses would diminish the internal reliability by the independent nature of random responses, but what it means by substantial and such an effect in general are less certain.

In practice, inconsistent responses may not be easily identified since they can also be plausible responses. Random responses are particularly difficult to detect as they have no identifiable patterns. Nevertheless, there are tested personality scales, namely, the Minnesota Multiphasic Personality Inventory-2 (MMPI-2) and the Minnesota Multiphasic Personality Inventory-Adolescent (MMPI-A), that assess the level of inconsistency for a response [4,5]. Both of them have a variable response inconsistency (VRIN) scale for assessing random responding and a true response inconsistency (TRIN) scale for assessing fixed responding. Cutoff values have also been established for the detection of random and fixed responses [4-6]. Depending on the instrument used, the VRIN scale comprises at least 50 item pairs and the TRIN at least 23 item pairs. As their length does not always allow for concurrent use with PRO instruments, we can only assess the sensitivity of internal reliability within an anticipated range of the proportion of inconsistent responses. However, to the best of our knowledge, no method is available for adjusting the internal reliability due to inconsistent responses.

In view of these, we aimed 1. to evaluate the impact of inconsistent responding on internal reliability; 2. to propose a method for adjusting Cronbach's alpha in the presence of inconsistent responses; and 3. to illustrate the use of the procedure in evaluating the internal reliability of the standard Chinese 12-item Short Form Health Survey (SF-12v2) for a large sample of adolescents.

## Methods

### Adjusting Cronbach's alpha for inconsistent responses

We consider a multi-item scale when the total score S is used as a health indicator. Cronbach's alpha requires adjustment when there are inconsistent responses. This could be done when the proportions of random and fixed responses, denoted by p_R _and p_F_, respectively, are known. Given these proportions, Cronbach's alpha based on the true responses (α_T_) can be derived as the following formula:(1)

where α is Cronbach's alpha without the adjustment for inconsistent responses, and m is the number of items. The quantities a and b are obtained from the equations:(2)

and(3)

when all items have the same polarity. μ_R _and  are the mean and variance of the random responses and can be taken as  and , respectively, for scales composing of items responded on a K-point Likert scale with each scored from 1 to K. μ_T _is the mean of true responses and can be taken as  [see Additional file 1].

Cronbach's alpha adjusted for inconsistent responding can be calculated from (1) after replacing the unknown quantities by the corresponding sample estimates. Note the adjustment assumes that both random and fixed responses to an item are uniformly distributed over the K-point Likert scale; i.e., there is no specific preference of a certain response category. Performance of the adjustment procedure is assessed by a small Monte-Carlo simulation study. Biases of the adjusted Cronbach's alpha are consistently smaller than those of the unadjusted alpha [see Additional file 2].

### Assessing the impact of inconsistent responses on Cronbach'salpha

The impact of inconsistent responses as well as the number of items and item response categories on Cronbach's alpha is analytically assessed by using our derived formula in (1). The assessment is performed under the following four settings that were chosen to cover some common scenarios in practice:

1. The influence of random responses is assessed by varying its proportion (p_R_) from 0 to 50% when p_F _is taken to be 0 or 5%. The mean difference between the true and random responses (μ_T_-μ_R_) is 0 or 1, and the scale has 5 positive polarity items, each responded on a 5-point Likert scale.

2. The influence of fixed responses is assessed by varying its proportion (p_F_) from 0 to 50%. The p_R _is taken to be 0 or 10%, and the number of positive polarity items is 5 or 3. Moreover, the mean difference between the true and random responses (μ_T_-μ_R_) is 0, and the scale has 5 items, each responded on a 5-point Likert scale.

3. The influence of the number of items is assessed by varying it from 2 to 20 when the proportion of positive polarity items is taken to be 0.5 or 1, and the mean difference between the true and random responses (μ_T_-μ_R_) is 0 or 1. Moreover, all items are responded on a 5-point Likert scale.

4. The influence of the number of item response categories (K) is assessed by varying it from 2 to 10 when the number of positive polarity items is 5 or 3, and the mean difference between the true and random responses is 0 or 0.2 K. Moreover, we assume that 20% and 5% of responses are random and fixed, respectively.

For each of the four scenarios, Cronbach's alpha based on the true responses is defined to be 0.4, 0.5, 0.6, 0.7 and 0.8.

### A real example to illustrate the adjustment of inconsistent responses

As an example, we evaluate the internal reliability of the standard Chinese SF-12v2. The questionnaire consists of 12 items in eight scales. For the sake of illustration, we considered only the Physical functioning (PF), Role emotional (RE) and Mental health (MH) scales, each of which consists of two items. All items in the three scales are positively worded except one item in MH that is negatively worded. Items in the PF scale use a 3-point Likert scale, while the other items use a 5-point Likert scale. The original scale scores are standardized in the range of 0-100, but for convenience, we just considered the total score after reverse coding the responses of the negative polarity items. Note, however, that the internal reliability is invariant to this standardization.

Data in the standard Chinese SF-12v2 were collected from the Hong Kong Student Obesity Surveillance (HKSOS) project conducted in 2006-2007. This study was cross-sectional involving 42 high schools covering all 18 districts in Hong Kong. It administered a survey questionnaire that contained the SF-12v2. The project was approved by the Institutional Review Board of The University of Hong Kong and the Hospital Authority Hong Kong West Cluster.

## Results

### The impact of inconsistent responses on Cronbach's alpha

**Figure 1:**
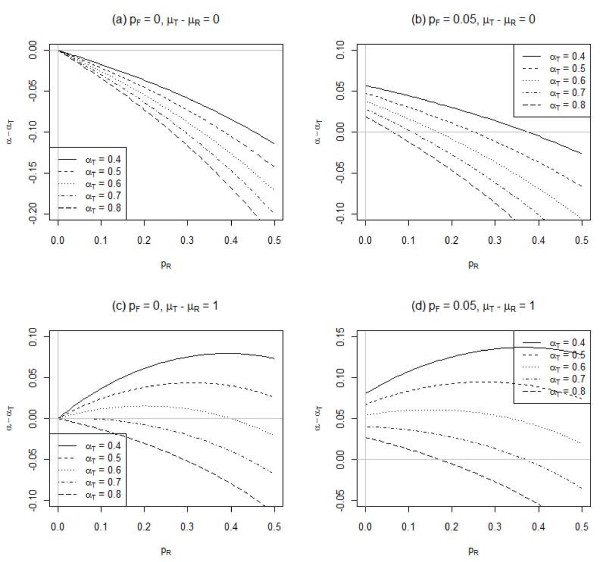
**Influence of random responses (p_R_) on Cronbach's alpha (α_T _for the true responses) for different percentages of fixed responses (p_F_) and mean differences between the true and random responses (μ_T_-μ_R_)**. There are 5 positive polarity items, each responded on a 5-point Likert scale.

Figure 1 shows the influence of random responses on the bias in Cronbach's alpha under setting 1. In general, the presence of random responses reduces the observed Cronbach's alpha (Figures 1(a) and 1(b)). In particular, when there are no fixed responses and the true responses are equal to the random responses on average, the reduction is more for higher Cronbach's alpha calculated from true responses. However, when the true responses are skewed relative to the random responses, Cronbach's alpha can be overestimated (Figures 1(c) and 1(d)). This is contrary to the common belief that the presence of random responses always reduces the internal reliability. The overestimation is higher when the true Cronbach's alpha is smaller.

**Figure 2:**
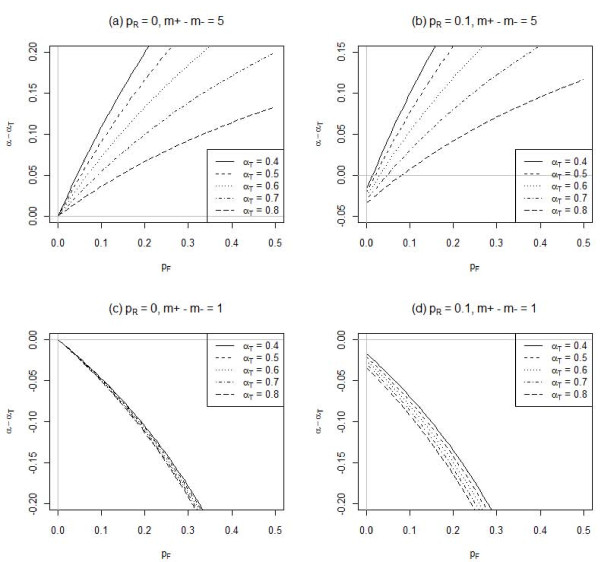
**Influence of fixed responses (p_F_) on Cronbach's alpha (α_T _for the true responses) for different percentages of random responses (p_R_) and numbers of positive (m_+_) and negative (m _-_) polarity items, m_+_-m _- _**. The mean random/fixed response is identical to that of the true responses, and there are 5 items, each on a 5-point Likert scale.

The influence of fixed responses under setting 2 is examined in Figure 2. The presence of fixed responses generally overestimates Cronbach's alpha when all items have the same polarity, but otherwise, it produces a smaller estimate. The bias is again higher when the true Cronbach's alpha is smaller.

**Figure 3:**
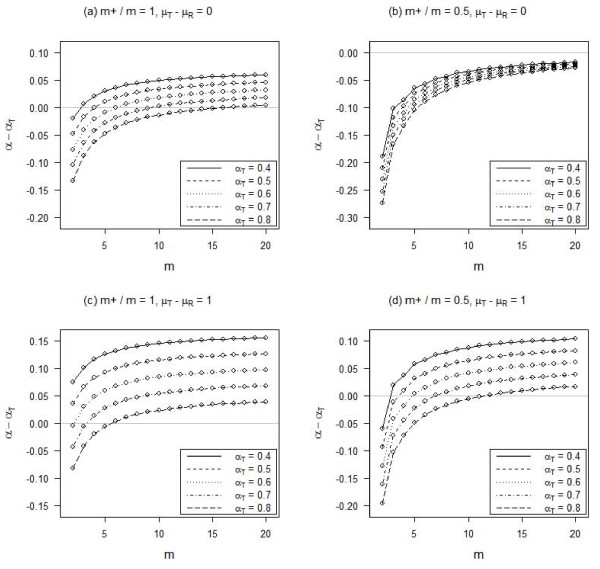
**Influence of the number of items (m) on Cronbach's alpha (α_T _for the true responses) for different proportions of positive polarity items (m_+_/m)**. There are 20% random responses and 5% fixed responses, no difference between the mean of the random/fixed responses and that of the true responses, and all items are responded on a 5-point Likert scale.

Figure 3 shows that the bias in Cronbach's alpha due to inconsistent responses may change from negative to positive with an increasing number of items under setting 3, but the effect of additional items beyond around 10 becomes small. On the other hand, a higher skewness of the true responses increases the differential in the bias under different true Cronbach's alpha levels (Figures 3(c) and 3(d)).

**Figure 4:**
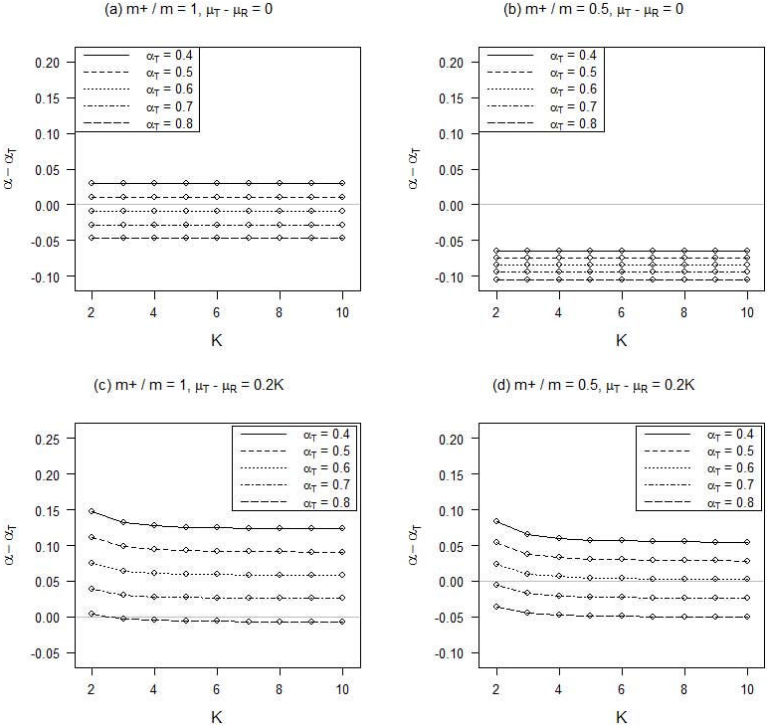
**Influence of the number of item response categories (K) on Cronbach's alpha (α_T _for the true responses) for different proportions of positive polarity items (m+/m) and mean differences between the true and random responses (μ_T_-μ_R_)**. There are 20% random responses and 5% fixed responses.

Under setting 4, the number of response categories does not generally have much influence on the bias of Cronbach's alpha due to inconsistent responses (Figure 4). There could be a small differential when there are only a few response categories and the true responses are skewed. However, the effect becomes smaller when there are more response categories.

### Internal reliability of the standard Chinese SF-12v2

We illustrate the adjustment of Cronbach's alpha due to inconsistent responses by evaluating the internal reliability of the standard Chinese SF-12v2. A total of 33,692 completed questionnaires from adolescents were received. A descriptive summary of the RE, PF and MH scales including their Cronbach's alpha coefficients are summarized in Table 1. Note the unusually low internal reliability of the MH scale, which may possibly be due to the presence of inconsistent responses. Although the survey questionnaire did not incorporate scales for tracking inconsistent responses, there were multiple response items other than those in the SF-12v2 with "none of the above" as a response choice. Random responses may be indicated if one or more responses were chosen simultaneously with the contradicting response of "none of the above". Using one to six such items closest to the SF-12v2, we estimated that there would be 1.5% to 11% of random responses in the SF-12v2. On the other hand, one item in the SF-12v2 consists of three sub-items about how often one feels 1. calm and peaceful, 2. energetic, and 3. downhearted and depressed. The same 5-point response scale from "all of the time" to "none of the time" was used. As the three sub-items are closely related and worded in different polarities, the selection of the same extreme response for all of them is suggestive of fixed responding. There were 4% of students who chose "all of the time" or "none of the time" in all three sub-items; this figure was doubled if the less extreme responses of "most of the time" and "a little of the time" were also counted. Hence, we estimated the percentage of fixed responses to be 4% to 8%. We shall now illustrate the adjustment of Cronbach's alpha for inconsistent responses. The adjusted Cronbach's alpha which is an estimate of α_T _is denoted by α_a_.

**Table 1:** A summary of scales of the standard Chinese SF12v2 in adolescents

	Scales		
	
	Role emotional (RE)	Physical functioning (PF)	Mental health (MH)
Number of response categories (K)	5	3	5
Number of items			
Positive polarity (m_+_)	2	2	1
Negative polarity (m_-_)	0	0	1
Number of respondents	32939	32924	33025
Mean	7.58	5.57	7.02
Variance	4.05	0.65	2.62
Floor^a^	2.2%	1.0%	0.6%
Ceiling^b^	25.7%	72.7%	6.7%
Cronbach's alpha	0.87	0.67	0.33

^a ^Percentage of values that equal to the plausible minimum of the scale

^b ^Percentage of values that equal to the plausible maximum of the scale

**Figure 5:**
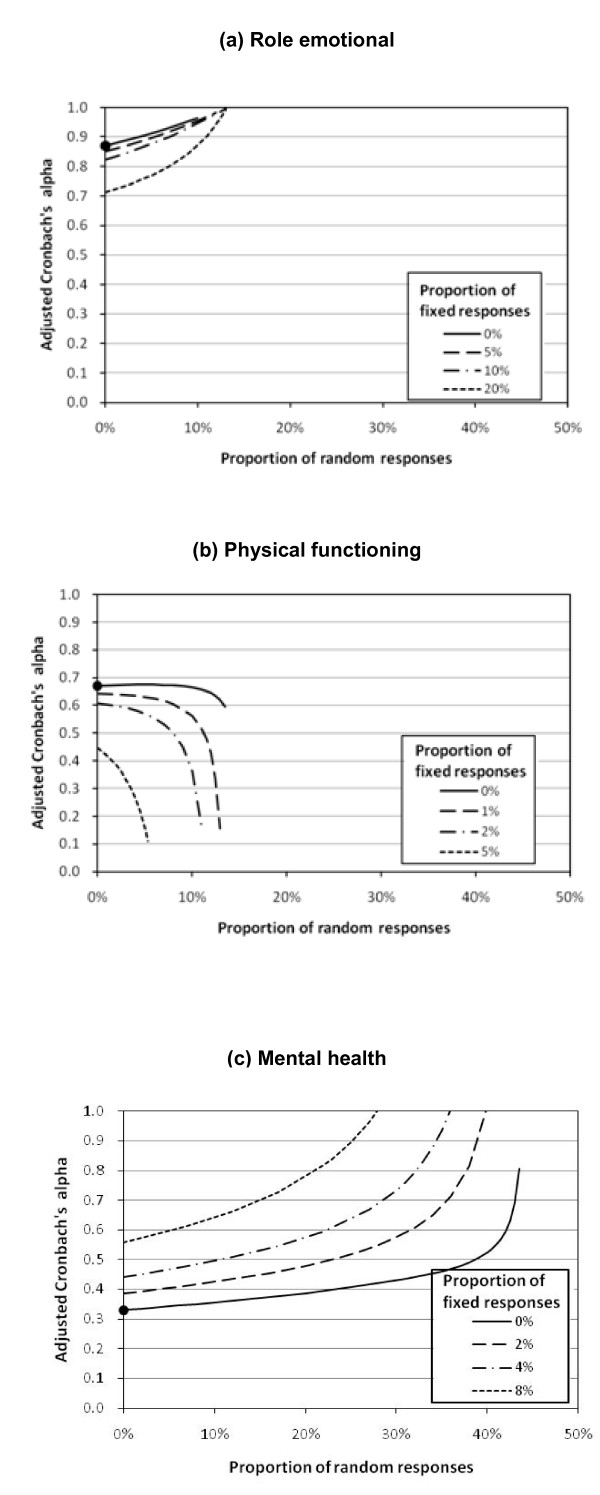
**Internal reliability of the standard Chinese SF-12v2 after removal of inconsistent responses**. The dot indicates Cronbach's alpha calculated when there are no inconsistent responses.

For the RE scale, K = 5, and thus μ_R _can be estimated as 3 and  as 2. When p_R _= 0.02 and p_F _= 0.05, we may estimate μ_T _as 3.850. By (2) and (3), we have a = 0.835 and b = 0.092. With α = 0.87, solving (1) yields α_a _= 0.868. The values of α_a _at other values of p_R _and p_F _are shown in Figure 5(a). The presence of random responses can reduce the internal reliability, and thus the true Cronbach's alpha can be underestimated. On the other hand, fixed responses inflate the observed association between the two positive polarity items and thus lead to over-estimation of the true Cronbach's alpha. Nevertheless, within our anticipated range of random and fixed responses, Cronbach's alpha for RE should be above 0.8. Therefore, the RE scale can be considered as internally reliable.

For the PF scale, K = 3, μ_R _is estimated as 2 and  as 0.67. The values of α_a _at different values of p_R _and p_F _are shown in Figure 5(b). While there remains an inflation of Cronbach's alpha when there are fixed responses, it is interesting to note a general decreasing trend of the true internal reliability after removing more random responses. In other words, the presence of random responses may also inflate Cronbach's alpha. A further examination of the scale items revealed that they were highly left skewed, with ceiling percentages of 80.4% and 81.5%, leading to 72.7% of the scale scores reaching the plausible maximum of 6 (Table 1). Indeed, random responses are systematically lower (μ_R_= 2) than true responses (μ_T_> 2). Thus, when there are random responses that uniformly spread over the plausible item values, small item values are more likely random responses than large item values. Consequently, individuals who gave random responses would more likely have small values in all items, and hence their presence would enhance the inter-item association. In fact, it can be shown that the presence of random responses increases the correlation between two positively worded items when the true correlation is below . This threshold increases when (μ_T_-μ_R_)^2^, which measures the skewness of the true responses from the mid-response, becomes large. In summary, the PF scale should have a Cronbach's alpha of at most 0.67 only, and its internal reliability could be unacceptably low given the anticipated range of inconsistent responses.

Figure 5(c) examines the impact of inconsistent responses on MH, which consists of a positive polarity and a negative polarity item. In contrast to the other two scales, the presence of both random and fixed responses would reduce the Cronbach's alpha of the MH scale. Thus, the reported Cronbach's alpha of 0.33 is indeed the minimum level, and the adjusted value could be as high as 0.66 given the anticipated range of inconsistent responses.

## Discussion

The presence of inconsistent responses may positively or negatively bias the Cronbach's alpha, making the assessment of internal reliability difficult. An adjustment was proposed to Cronbach's alpha for correcting the effects of inconsistent responses when one can estimate a possible range for the percentage of inconsistent responses. This enables a sensitivity analysis to assess the potential impact of inconsistent responses and facilitates a better understanding of the internal reliability of a multi-item scale.

As one would expect, the presence of fixed responses overestimates Cronbach's alpha for scales composed of items mostly worded in the same direction but would otherwise lead to an underestimation. However, it is interesting to observe that random responses may indeed inflate Cronbach's alpha when the distribution of true responses is skewed or, more precisely, when the true mean response deviates from the random/fixed mean response. This is contrary to the common intuition that random responses would dilute the association among items and hence reduce the internal reliability. Indeed, when the true item responses are skewed on the same side, the addition of random responses that scatter around the mid-response could strengthen association among the items if they are not too many. Thus, paradoxically, this kind of noise could inflate the internal reliability and hence Cronbach's alpha. Unfortunately, it is common for true responses to differ from random/fixed responses, on average, especially in patients whose quality of life has deteriorated due to their adverse conditions. Hence, we should be careful not to optimistically interpret Cronbach's alpha when there are random responses.

To determine random and fixed responses, tested personality scales such as the VRIN and TRIN scales of the MMPI-2 and MMPI-A may be considered [4]. They are, however, rather lengthy, requiring at least 23 item pairs, and they may not be feasibly incorporated into large scale studies. Nevertheless, we need to have an estimate of the proportion of inconsistent responses in a sample before the proposed method can be effectively applied. While the determination of whether an individual was endorsing inconsistent responses can be a challenge, modification or addition of a few items for tracking potentially inconsistent responses will be helpful. As in our illustrative example, the response option of "none of the above" in items allowing multiple response choices could be easily incorporated to track for potential random responses. Fixed responses are more easily identified by the patterns that they follow. Incorporating items that would not likely receive the same response will be useful.

Cronbach's alpha of a scale has been known to be higher in scales with more items [7]. We have found that, when there are inconsistent responses, scales with more items would also increase any upward bias in Cronbach's alpha. Although the increase diminishes and may become negligible when there are many items, it is better to keep the number of items minimal to avoid reporting an overly optimistic Cronbach's alpha. Nevertheless, there remains a chance of under-estimating Cronbach's alpha, and it is probably better to be conservative when assessing the internal reliability of a scale.

We have also shown that the number of response categories does not have much influence on the bias of Cronbach's alpha induced by the presence of inconsistent responses. There could be only a small positive increase in the bias for scales with items of 3 or fewer response categories. Previous studies have shown that scales with fewer response categories tend to have lower internal reliability and suggested the use of more than 3 response categories [8,9]. This recommendation is indeed also good to minimize the impact of inconsistent responses. However, the choice of the number of response categories may largely depend on the actual content of the scale [10]. Modern assessment of item characteristics utilizing item response theory is deemed more useful to determine an appropriate number of response categories [11].

We have illustrated how Cronbach's alpha can be adjusted for inconsistent responses by evaluating the standard Chinese SF-12v2 in a large sample of students. Note that each scale of the SF-12v2 consists of at most two items only. Although the Cronbach's alpha may in theory be used for scales of at least two items, its use for two-item scales has been criticized [12]. The concern lies in whether two items are sufficient to represent the correspondingly larger domain comprising a much larger collection of items. Alternative forms of reliability that utilize more items in the same construct may be more desirable [13]. Hence, the internal reliability of the SF-12v2 may require further study. It is used here to merely illustrate the impact of inconsistent responses on Cronbach's alpha.

The proposed adjustment to Cronbach's alpha for correcting the effects of inconsistent responses facilitates the assessment of the impact of inconsistent responses on internal reliability. In practice, as soon as respondents with inconsistent item-answer behavior had been identified, it would be simpler to exclude them from the calculation of Cronbach's alpha. However, when the identification of such responses is difficult and the anticipated range of inconsistent responses may be taken more conservatively than that of actually identified, the proposed adjustment may be used.

We assumed the random and fixed responses to an item are uniformly distributed over a K-point Likert scale. When an individual is endorsing a random or fixed response to an item without referencing to the actual content of the time, there would likely be no specific preference on endorsing a particular response category. Therefore, unless there are particular response categories that would be generally endorsed more often in the population, the assumption of uniform distribution appears to be reasonable. Nevertheless, a non-uniform distribution may also be incorporated. Indeed, the adjustment procedure depends on only the first two moments of the random and fixed responses. A different mean of random and fixed responses would either increase or decrease its difference from the mean of true responses (i.e. μ_T_-μ_R_), on which the influence has been examined in Figure 1. On the other hand, an increase of the variance of random and fixed responses would increase the proportion of variance in the total score that is due to inconsistent responses (i.e. /variance of S) which reduces the observed Cronbach's alpha.

We have not examined the impact of inconsistent responses on inference about Cronbach's alpha. However, it has been previously shown that the width of the corresponding confidence interval is negatively proportional to the estimated Cronbach's alpha [14,15]. Thus a positively biased alpha would tend to result in a short confidence interval leading to a nominal coverage less than the required level. Hence, the false positive error rate for testing about the significance of Cronbach's alpha would also be increased.

Cronbach's alpha has been criticized on the grounds that is just a lower bound of reliability and that other measures may be considered as a better lower bound measure than the coefficient alpha [16]. Moreover, it implicitly assumes the items are responded on an interval scale which limits its use in PRO instruments when items are categorically scored. Besides, it assumes a fixed level of reliability across the whole range of the measurement, and is not a measure of uni-dimensionality. Nevertheless, Cronbach's alpha may be interpreted as a measure of the proportion of the total score variance that can be attributed to true score variance that may be affected by the extent to which the items are associated. Hence, we believe that the impact of inconsistent responses could be applicable to the general evaluation of internal reliability of a scale. An analytical exploration of the impact of inconsistent responses would be desirable. A potential method was the modern psychometric assessment by item response theory which allows the examination of the response characteristics of individual items. It has gained much popularity but it has been reviewed and concluded to be relatively unsuccessful in identifying dissimulation [17,18]. Further work may deem to be necessary.

## Conclusions

Cronbach's alpha may be inflated by inconsistent responses when either the mean of true responses differ from that of the random/fixed responses or all items in the scale have the same polarity. The inflation in the former situation is due to the presence of random responses, while the latter is due to the presence of fixed responses. It should not be assumed that random responses always diminish Cronbach's alpha.

## Competing interests

The authors declare that they have no competing interests.

## Authors' contributions

DYTF contributed to the methodological development, data analysis and drafting of the manuscript. SYH and THL critically revised the manuscript. All authors have read and approved the final manuscript.

## Supplementary Material

Additional file 1 **Derivation of the Cronbach's alpha for true responses when there are inconsistent responses**. It describes in details about the derivation of the Cronbach's alpha for true responses when there are inconsistent responses. Click here for file

Additional file 2 **A Monte-Carlo simulation study**. It describes details of a Monte-Carlo simulation study and shows the corresponding results. Click here for file
