# Supplementary material for: Evaluation of internal reliability in the presence of inconsistent responses
Source: Health Qual Life Outcomes. 2010 Mar 12;8:27. doi: 10.1186/1477-7525-8-27 (PMC2842254; doi:10.1186/1477-7525-8-27)
Supplement: Additional file 1 — Derivation of the Cronbach's alpha for true responses when there are inconsistent responses. It describes in details about the derivation of the Cronbach's alpha for true responses when there are inconsistent responses. [file 1477-7525-8-27-S1.RTF]

Additional file 1
Derivation of the Cronbach's alpha for true responses when there are inconsistent responses
We consider a scale consisting of  items ( are positively worded, and  are negatively worded), each responded on a -point Likert scale taking values from 1 to. The total of item responses after reverse coding of the negatively worded items is used as a health indicator. For a respondent, let  be the response to the  item  after reverse coding where necessary. Then, the Cronbach's alpha is
		
for assessing the internal reliability of the scale.

To account inconsistent responses, let  be an indicator of the type of response  endorsed by an individual and  = or when  is a true, random or fixed response, respectively. Then, if ,  and  are the probabilities for a response being true, random and fixed, respectively such that , the probability mass function of  is 
.
As random responses are given unsystematically, they are taken as independently and identically distributed with . For fixed responses, the same response is given to all items and thus  for any  Note the calculation of the Cronbach's alpha does not require full distributional specification of  but only their first two moments, denoted by  ,  and  depending on the value of . We assume the chance for the endorsement of a random or a fixed item response is the same, and . This is not an unrealistic assumption as there should be no specific preference on the responses if the content of the item is not referenced. Note, however, this assumption can be relaxed but its application simplifies the calculation of the Cronbach's alpha. 

In the presence of inconsistent responses, the Cronbach's alpha can be obtained by first deriving  and  in (1).   can be written as
 
= 
	 
	 
=	, taking  and . is the correction term when there are items of different polarity which equals .
Similarly,  equals

=	
	 
	 
=	 
	 
	 
after taking  and .
Substituting them into (1) gives
		
where  is the Cronbach's alpha based on true responses, , and. To obtain  and , we need to first specify the proportions of random and fixed responses as well as their first two moments. The remaining parameters  and  can then be solved by
	 and	


		
Note when all item have the same polarity, (4) reduces to 
	 
.
Hence, the internal reliability for the true responses may be evaluated at different percentage of inconsistent responses. Note when there are no inconsistent responses, i.e. = 0 and = 1, we can deduce from (4) that . By which, we can work out  = 1 and  = 0, and thus  from (2). 
