# Supplementary material for: Evaluation of internal reliability in the presence of inconsistent responses
Source: Health Qual Life Outcomes. 2010 Mar 12;8:27. doi: 10.1186/1477-7525-8-27 (PMC2842254; doi:10.1186/1477-7525-8-27)
Supplement: Additional file 2 — A Monte-Carlo simulation study. It describes details of a Monte-Carlo simulation study and shows the corresponding results. [file 1477-7525-8-27-S2.RTF]

Additional file  2
A Monte-Carlo simulation study
We considered a scale of 10 items with each item responded on a 5-point Likert scale and completed by 1000 individuals. At total of 1000 samples were generated under a fixed choice of the proportion of random responses (taken as either 0%, 10% or 30%), proportion of fixed responses (taken as either 0%, 10% or 30%), probability distribution of the true responses for the first item (taken as either (0.1, 0.2, 0.4, 0.2, 0.1) or (0.1 0.1, 0,2, 0.2, 0.4)), and a r value that was used to induce association among the true item responses. Specifically, true responses for items other the first one were taken as *r where  is the generated true response for the first item and x is generated independently from the same probability distribution of the true response for the first item.  For each generated sample, Cronbach's alpha with and without the adjustment for inconsistent responses as well as the alpha based on only the true responses were calculated.  The biases of the unadjusted and adjusted alphas were obtained by subtracting the true Cronbach's alpha from them and taking the average over the 1000 generated samples.  Results of the simulation study are shown in Table A.1.


Table A.1.  Biases of the unadjusted and adjusted Cronbach's alpha in a Monte Carlo simulation study
(a)	Probability distribution of the true response for the first item = (0.1, 0.2, 0.4, 0.2, 0.1)
Percentage of random responses	Percentage of fixed responses	Mean of  based on true responses	Bias of unadjusted 	Bias of adjusted 	
0%	10%	0.37 	0.438 	0.206 	
0%	10%	0.70 	0.201 	-0.010 	
0%	10%	0.86 	0.090 	-0.008 	
0%	30%	0.38 	0.557 	0.405 	
0%	30%	0.70 	0.261 	0.066 	
0%	30%	0.86 	0.123 	-0.014 	
10%	0%	0.38 	0.199 	-0.003 	
10%	0%	0.70 	0.096 	-0.002 	
10%	0%	0.86 	0.021 	-0.002 	
10%	10%	0.38 	0.440 	0.213 	
10%	10%	0.70 	0.198 	-0.010 	
10%	10%	0.86 	0.078 	-0.009 	
10%	30%	0.37 	0.553 	0.406 	
10%	30%	0.70 	0.251 	0.081 	
10%	30%	0.86 	0.110 	-0.014 	
30%	0%	0.37 	0.285 	0.013 	
30%	0%	0.70 	0.122 	-0.012 	
30%	0%	0.86 	0.015 	-0.006 	
30%	10%	0.37 	0.430 	0.240 	
30%	10%	0.70 	0.180 	0.009 	
30%	10%	0.86 	0.053 	-0.023 	
30%	30%	0.37 	0.528 	0.405 	
30%	30%	0.70 	0.226 	0.106 	
30%	30%	0.86 	0.084 	0.000 	


(b)	Probability distribution of the true response for the first item = (0.1, 0.1, 0.2, 0.2, 0.4)
Percentage of random responses	Percentage of fixed responses	Mean of  based on true responses	Bias of unadjusted 	Bias of adjusted 	
0%	10%	0.47	0.434 	0.239 	
0%	10%	0.64	0.296 	0.090 	
0%	10%	0.84	0.113 	-0.017 	
0%	30%	0.47	0.498 	0.355 	
0%	30%	0.64	0.340 	0.195 	
0%	30%	0.84	0.140 	0.010 	
10%	0%	0.47	0.324 	0.009 	
10%	0%	0.64	0.211 	-0.005 	
10%	0%	0.84	0.053 	-0.002 	
10%	10%	0.47	0.439 	0.259 	
10%	10%	0.64	0.293 	0.109 	
10%	10%	0.84	0.103 	-0.019 	
10%	30%	0.47	0.492 	0.361 	
10%	30%	0.64	0.329 	0.207 	
10%	30%	0.84	0.129 	0.019 	
30%	0%	0.47	0.379 	0.155 	
30%	0%	0.64	0.240 	0.025 	
30%	0%	0.84	0.055 	-0.010 	
30%	10%	0.47	0.429 	0.284 	
30%	10%	0.64	0.276 	0.142 	
30%	10%	0.84	0.083 	-0.016 	
30%	30%	0.47	0.469 	0.367 	
30%	30%	0.64	0.308 	0.224 	
30%	30%	0.84	0.105 	0.039 	
